# ALS Mutations Shift the Isoelectric Point of the KIF5A C Terminal Inducing Protein Aggregation and TDP-43 Mislocalization

**DOI:** 10.1523/JNEUROSCI.1658-24.2025

**Published:** 2025-06-24

**Authors:** Pietro Zanella, Isabel Loss, Rosanna Parlato, Jochen H. Weishaupt, Carlo Sala, Chiara Verpelli, Tobias M. Boeckers, Alberto Catanese

**Affiliations:** ^1^German Center for Neurodegenerative Diseases (DZNE), Ulm site, Ulm 89081, Germany; ^2^Institute of Anatomy and Cell Biology, Ulm University, Ulm 89081, Germany; ^3^Division of Neurodegeneration, Department of Neurology, Medical Faculty Mannheim, Mannheim Center for Translational Neurosciences, Heidelberg University, Mannheim 68167, Germany; ^4^Department of Neurology, Ulm University, Ulm 89081, Germany; ^5^CNR, Neuroscience Institute, Vedano al Lambro 20854, Italy; ^6^Department of Neuroanatomy, Institute of Anatomy and Cell Biology, Freiburg 79104, Germany

**Keywords:** aggregation, ALS, isoelectric point, KIF5A, TDP-43

## Abstract

Amyotrophic lateral sclerosis (ALS) is a devastating neurodegenerative disease characterized by death of lower and upper motor neurons. Although the mechanism behind the selective neuron loss is still unclear, several heterogeneous genes have been causally linked to ALS. *KIF5A* encodes for a neuronally enriched kinesin involved in protein transport, and mutations within this gene have been causally linked to different motor neuron diseases. The mutations identified in ALS patients are mostly predicted to alter its mRNA splicing, leading to a frameshift mutation and an aberrant 39-aa-long sequence in the C-terminal domain of KIF5A. Here we found that ALS-related KIF5A mutations induce the accumulation of the mutant form of the protein in human motoneurons, which are also characterized by the cytosolic mislocalization of TDP-43. This ALS hallmark was even exacerbated upon overexpression of the ALS-KIF5A protein in cells differentiated from healthy controls and primary neurons, suggesting a pathological connection between the cellular load of the mutant protein and TDP-43 pathology. While the terminal domain of the WT isoform is characterized by an acid isoelectric point (pI), the ALS variant presents a basic pI due to the altered aminoacidic composition of this sequence. We thus generated a KIF5A-ALS isoform that retained part of the aberrant sequence but with lower pI. The overexpression of this mutated variant led to significantly lower protein aggregation and TDP-43 mislocalization than the ALS mutant. Our data show that re-establishing the correct pI rescues KIFA aggregation and significantly reduces the cytoplasmic mislocalization of TDP-43.

## Significance Statement

Amyotrophic lateral sclerosis is a lethal neurodegenerative disease to which no cure is still known. Heterogeneous genes have been causally linked to ALS, yet, the exact pathomechanism responsible for neuronal death remains unclear. One such gene is KIF5A which encodes for a neuronally enriched kinesin. Identified mutations cause incorrect mRNA splicing resulting in an aberrant C-terminal aminoacidic sequence. Here, we identified TDP-43 cytosolic enrichment, a hallmark common to many ALS models, in two distinct hiPSC-derived motoneuron lines harboring the ALS mutation KIF5A^c2993-1 G>A^. Moreover, we generated a KIF5A isoform that retained most of the aberrant sequence but did not promote protein aggregation or TDP-43 mislocalization upon overexpression. These results shed further light on the pathobiochemistry of the ALS-KIF5A cases.

## Introduction

Amyotrophic lateral sclerosis (ALS) is a devastating neurodegenerative disease mainly characterized by the loss of upper and lower motoneurons. Although the mechanisms behind this selective neuronal vulnerability are still not completely clear, several genes with heterogeneous functions have been causally linked to this pathology. One such gene is *KIF5A*, which encodes a neuronally enriched kinesin involved in anterograde transport of among others, protein complexes, and cellular organelles such as mitochondria and RNA granules (Vale et al., 1985; Aizawa et al., 1992; Hirokawa, 2006).

The *KIF5A* mutations identified in ALS patients are mostly predicted to alter its mRNA splicing, leading to a pathological frameshift affecting the cargo binding domain (Brenner et al., 2018; Nicolas et al., 2018; Baron et al., 2022; Pant et al., 2022). As a consequence of this frameshift, recent studies have shown that ALS mutations lead to the generation of an aberrant, 39-aa-long sequence in the C-terminal domain. Interestingly, the presence of this mutated C terminal appears to be sufficient to induce the aggregation of KIF5A, indicating a toxic gain-of-function as pathological mechanism specifically characterizing these ALS cases (Nakano et al., 2022). In fact, mutations occurring within the motor and coiled-coil domains of the protein (located at the N terminal) are causally associated with hereditary spastic paraplegia type 10 (SP10) and Charcot–Marie–Tooth disease type 2 (CMT), two nonfatal neuropathies (Xia et al., 2003; Fichera et al., 2004). These have also been causally linked to mutations in other kinesins such as KIF1A, KIF1B (Zhao et al., 2001; Chiba et al., 2019; Boyle et al., 2021; Budaitis et al., 2021), and DYNC1H1 (Weedon et al., 2011). Since the mortality of CMT and SP10 patients is significantly lower than ALS, it is reasonable to speculate that the toxic aggregation of KIF5A might even contribute to the severity of the disease affecting individuals with C-terminal mutations in this gene. The accumulation of toxic protein aggregates is indeed a common feature of fatal neurodegenerative disorders, and in the specific case of ALS the pathological cytoplasmic accumulation of TDP-43 represents a common hallmark characterizing the vast majority of cases. This implies a crucial contribution to neuronal sufferance and pathology progression played by these aberrant structures. In fact, the transsynaptic transfer of cytotoxic protein aggregates has been suggested to contribute to the pathology spreading across the central nervous system in the case of different neurodegenerative diseases, including ALS (Braak et al., 2013).

For these reasons, a better understanding of the (patho)biochemical properties characterizing the aberrant protein aggregates observed in most of the neurodegenerative diseases is needed in order to contrast their toxic effects on vulnerable neurons. Cytotoxic accumulation of KIF5A aggregates affects axonal transport and synaptic activity, eventually leading to cellular death (Pant et al., 2022; Soustelle et al., 2023). However, the biochemical properties of the aberrant C terminal linked to the ALS mutations are only partially characterized. Accordingly, the biochemical impact of this aberrant sequence on protein aggregation, localization, solubility, and toxicity is still controversial (Rich et al., 2023; Soustelle et al., 2023; Cozzi et al., 2024; Layalle et al., 2024).

In this study, we demonstrate that the aggregation of KIF5A is directly linked to a drastic shift of the protein's isoelectric point in the presence of the aberrant 39 aa sequence. We also provide evidence indicating that the aggregation behavior of the mutant KIFA depends on its expression level and is sufficient to trigger the cytoplasmic mislocalization of TDP-43. In conclusion, our work provides new insights into the KIF5A pathobiochemistry linked to ALS and reveals novel pathological aspects linked to these specific mutations.

## Material and Methods

### Human iPSCs

Human keratinocytes derived from ALS patient's hair were reprogrammed to generate the KIF5A-ALS hiPSC line (KIF5A^c2993-1^ I), as previously described (Takahashi and Yamanaka, 2006; Aasen et al., 2008; Linta et al., 2012). Keratinocytes at 75% confluence were treated with 5 × 10^5^ proviral genome copies for two subsequent days and transferred to previously irradiated (30 Gy) rat embryonic fibroblasts feeder cells. Keratinocytes were kept in a 5% O_2_ atmosphere and cultured in hiPSC medium [KnockOut DMEM, 20% KnockOut Serum Replacement, 2 mM GlutaMAX, 100 mM MEM nonessential amino acids (NEAA), 1% antibiotic–antimycotic, 100 mM β-mercaptoethanol, 50 mg/ml vitamin C, and 10 ng/ml FGF-2]. After 15–20 d of cultivation, it was possible to detect hiPSCs growing in colonies with the characteristic morphology. Colonies were mechanically detached and further cultivated on feeder-free plates using mTeSR plus medium (STEMCELL Technologies, 100-0276).

The isogenic control (KIF5A^WT^ I) was generated with CRISPR by Synthego correcting the patient mutation. Briefly, patient's hiPSC were transfected by electroporation with Cas9 complexed with the guide RNA (CCUAUUGUCAUUGAUAUCUG) and the donor DNA (ATCTCCTTTTTTCTTCTTCTAATCCTGTGTTCTCAATGATGATCTCTTCAGGAAATGCAACAGATATCAATGACAATAGGTACAACAGTCCCCACTACCCC) together. After 2 d, the percentage of knock-in sequences in the edited cell pool was assessed by PCR amplification (primer forward: CCTTTAGGTCTCAGGCTGCC, primer reverse: ATGAGTCTCACTCCCTCCCC) and Sanger sequencing of the targeted site. The edited cell pool was used to seed single cells for clonal expansion. Every cell population was regularly controlled to ensure that it was the progeny of one single cell. After clonal isolation and sanger sequencing, two clones were successfully selected from the cell pool. Genomic DNA of both cell lines was sequenced to exclude chromosomal aberration and to ensure that the patient heterozygous KIF5A variant (KIF5A^c2993-1 G>A^) was maintained in the ALS cell line while the mutation was corrected in the isogenic control.

The second ALS cell line (KIF5A^c2993-1^ II) was generated inserting the c2993-1 G > A mutation in the commercial KOLF2.1J cell line (JIPSC001000, Jackson Laboratory), henceforth called KIF5A^WT^ II, by CRISPR using guide RNA (UUCUCAAUGAUGAUCUCUUCAGG) and donor DNA (AGAGAACCCAGGGGTAGTGGGGACTGTTGTACCTATTGTCATTGATATCTGTGGCATTTCTTGAAGAGATCATCATTGAGAACACAGGATTAGAAGAAGA). Heterozygosity of the resulting clones was assessed with PCR amplification (primer forward: TGGACAATGGTGAGTGAAAAAGATG, primer reverse: TACAGGAATACAGGGAAACTCAAGG) and Sanger sequencing.

HiPSCs were cultured at 37°C (5% CO_2_, 5% O_2_) on Matrigel-coated (Corning, 354277) 6-well plates using mTeSR Plus medium (STEMCELL Technologies, 100-0276). At 80% confluence, colonies were detached using Dispase (STEMCELL Technologies, 07923) and passaged in a 1:3 or 1:6 split ratio. Cells were routinely controlled for Mycoplasma contamination with MycoStrip—Mycoplasma Detection Kit (Invivogen, rep-mysnc-50) and Mycoplasma PCR Detection Kit (ABM, G238).

### Differentiation of hiPSC-derived motoneurons

Neuronal differentiation was carried out as previously described (Catanese et al., 2021). hiPSC colonies were detached and cultivated in suspension in hESC medium (DMEM/F12, 20% KnockOut Serum replacement, 1% NEAA, 1% β-mercaptoethanol, 1% antibiotic–antimycotic, 10 µM SB-431542, 1 µM dorsomorphin, 3 µM CHIR 99,021, 1 µM purmorphamine, 200 ng/µl ascorbic acid, 1% B27, 0.5% N2) in ultralow attachment flasks T75 for 3 d to form embryoid bodies (EBs). On the fourth day, the medium was switched to MN Medium (DMEM/F12, 24 nM sodium selenite, 16 nM progesterone, 0.08 mg/ml apotransferrin, 0.02 mg/ml insulin, 7.72 µg/ml putrescine, 1% NEAA, 1% antibiotic–antimycotic, 50 mg/ml heparin, 10 µg/ml of the neurotrophic factors BDNF, GDNF, and IGF-1, 10 µM SB-431542, 1 µM dorsomorphin, 3 µM CHIR 99,021, 1 µM purmorphamine, 200 ng/µl ascorbic acid, 1 µM retinoic acid, 1 µM cAMP, 1% B27, 0.5% N2). After 5 further days of cultivation, EBs were dissociated into single cells with Accutase (Sigma-Aldrich, A6964) and plated onto μDishes, 24-well µPlates (Ibidi) or 12-well plates (Sarstedt) precoated with Growth Factor Reduced Matrigel (Corning, 356,231) in Diff4 medium (DMEM/F12, 24 nM sodium selenite, 16 nM progesterone, 0.08 mg/ml apotransferrin, 0.02 mg/ml insulin, 7.72 µg/ml putrescine, 1% NEAA, 1% antibiotic–antimycotic, 50 mg/ml heparin, 10 µg/ml of the neurotrophic factors BDNF, GDNF, and IGF-1, 1 µM purmorphamine, 200 ng/µl ascorbic acid, 1 µM retinoic acid, 1 µM cAMP, 2% B27). Neurons were kept in culture at 37°C (5% CO_2_) changing half medium twice a week.

To overexpress KIF5A constructs in motoneurons, Lipofectamine 3000 Transfection kit (Thermo Fisher Scientific, L3000001) was used. Briefly, DNA was mixed with P3000 reagent in DMEM and subsequently with Lipofectamine 3000 for 15 min at room temperature. The solution was added to DIV19 motoneurons. Cells were fixed 2 d after transfection.

### Antibodies

A custom-made antibody selective for the ALS isoform of KIF5A was purchased from Pineda (Germany). This polyclonal antibody was purified by chromatography affinity from the serum of rabbits immunized for 2 months against a peptide with sequence: “KRQQPANLPHPRLHTC”, corresponding to ALS isoform sequence from amino acid 1018 to 1034. All the antibodies used in this paper are listed in Table S1.

### RIPA-Urea fractionation

Collected neurons were resuspended in cold RIPA buffer supplemented with protease and phosphatase inhibitor and sonicated (5 cycles of 10 s). Whole cell extracts were then centrifuged 15,000 rpm for 15 min at 4°C. The supernatant, containing the RIPA soluble fraction, was collected in a cold centrifugation tube while the pellet was washed three times with RIPA buffer and dissolved in a buffer containing Urea 8 M (10 mM Tris-HCl, 8 M Urea, pH 8, protease and phosphatase inhibitor in ultrapure water). Protein concentration was determined by Pierce BCA Protein Assay Kits (Thermo Fisher Scientific, 23225), and 10 µg of protein was resolved on TGX Stain-Free FastCast Acrylamide 7.5% gel (Bio-Rad, 1610180).

### Western blots

Collected cells were resuspended in cold RIPA buffer and sonicated (five cycles of 10 s). After 1 h of rotation at 4°C, samples of equal concentration of protein, determined by Pierce BCA Protein Assay Kits (Thermo Fisher Scientific, 23225), were denatured in Laemmli sample buffer (Bio-Rad, 1610747) for 15 min at 95°C. Samples were run on hand-cast polyacrylamide SDS-PAGE gels (Serva, 10687.01) and then transferred to a nitrocellulose membrane using a Trans-Blot Turbo device (Bio-Rad). To block nonspecific binding sites, the membranes were incubated with a 5% BSA (Sigma-Aldrich, A9647) or 5% skim milk (Millipore, 115363) solution diluted in TBST (TBS buffer pH 7.5 + 0.2% Tween) for 1 h and incubated with the primary antibody overnight at 4°C. Membranes were washed three times with TBST, incubated with HRP-conjugated secondary antibody for 2 h at RT, and again washed three times. Chemiluminescent signal was detected using the ECL detection kit (Thermo Fisher Scientific, 32106) and a MicroChemi 4.2 device (DNR Bio Imaging System). After acquisition, membranes were washed again and stripped with a 2 M NaOH solution for 3 min. Membranes were equilibrated for 30 min in TBST and incubated with new antibodies. For quantification, Gel Analyser Software 2010a was used.

### qRT-PCR

Total RNA was extracted from hiPSC-derived MN using the RNeasy Mini kit (Qiagen) following the manufacturer's instructions. First-strand synthesis and quantitative real-time PCR amplification were performed in a one-step using the QuantiFast SYBR Green RT-PCR kit (Qiagen) in a total volume of 20 µl. QuantiTect Primers validated without sequence information directed against the genes indicated within the text were purchased from Qiagen. The PCR was set as follows: initial reverse transcription step 10 min at 55°C and a first denaturation step (5 min at 95°C), followed by 40 cycles of denaturation (5 s at 95°C), one-step annealing and elongation (10 s at 60°C). SYBR Green I reporter dye's signal was measured against the internal passive reference dye (ROX) to normalize non-PCR-related fluctuations. Rotor-Gene Q software (version 2.0.2) was used to calculate the cycle threshold values.

### Mutagenic PCR

KIF5A constructs were generated by site-direct mutagenesis using Phusion Site-Directed Mutagenesis Kit (Thermo Fisher Scientific, F541). Mutagenesis was performed following manufacturer's guide and adding DMSO 3% to the reaction mix. Primers, templates, and temperatures of annealing are listed in Table 1 and Table S2. PCR products were purified with GeneJET PCR Purification Kits (Thermo Fisher Scientific, K0701) and resuspended in DNAse-free water (Carl Roth, EG-Nr. 231-791-2).

**Table 1.** Sequence of the primers used in this paper

Primer name	Sequence
KIF5A var1 Fw	CAG CAG CCA GCT AAT CTC CCA CAC
KIF5A var1 Rv	GCA GGT CAC TCC ATT GTC CAT GTT
KIF5A var1.2 Fw	TCC CAC ACC CAG AGC TGC ATA CC
KIF5A var1.2 Rv	GAT TAG CTG GCT GCT GGC AGG TCA
KIF5A var1.3 Fw	CCC ACG GCT GGA TAC CTG CAC TT
KIF5A var1.3 Rv	TGT GGG AGA TTA GCT GGC TGC TGG
KIF5A var2 Fw	CCA CAC CCA GAG CTG CAT ACC TG
KIF5A var2 Rv	GAG ATT AGC TGG CTG CTG TCT CTT GG
KIF5A var2.3 Fw	CCA CAC CCA GAG CTG GAT ACC TG
KIF5A var3 Fw	CCC ACG GCT GGA TAC CTG CAC TT
KIF5A var3 Rv	TGT GGG AGA TTA GCT GGC TGC TGT C
KIF5A var4a Fw	AGG CTG AGG ACC AGG CCA AGC TTT T
KIF5A var4a Rv	TCC ATT GTC CAT GTT GGC CTT CTG G
KIF5A var4 Fw	TAA GAA TTC TGC AGA TAT CCA GCA CAG TGG C
KIF5A var4 Rv	ATG CAG CCG TGG GTG TGG GAG ATT

### *E. coli* transformation

Purified PCR products were used to transform *E. coli* DH5α strain (Thermo Fisher Scientific, 18258012) by electroporation. After incubation in SOB medium (20 mg/ml tryptone, 5 mg/ml yeast extract, 0.5 mg/ml NaCl, 250 mM KCl) for 1 h at 37°C, transformed *E. coli* were plated in LB agar (Carl Roth, X969.3) petri dish containing ampicillin 1:1,000 (Carl Roth, K029.2) as selection marker. After 16 h of incubation at 37°C, single colonies were manually picked and incubated in LB medium (Carl Roth, X968.3) containing ampicillin for 6 h at 37°C. Plasmids were purified using QIAprep Spin Miniprep Kit (Qiagen, 27104) and resuspended in DNAse-free water. Correct mutation in the constructs was ensured upon sequencing (Eurofins Genomics).

To expand the title of the purified plasmid, *E. coli* (DH5α strain) was electroporated with 10 ng of the purified constructs. Transformed *E. coli* was selected in LB agar with ampicillin, and manually picked colonies were cultured for 16 h at 37°C in LB medium with ampicillin. Plasmids were finally purified using NucleoBond Xtra Midi Kit (Macherey-Nagel, 740410.5) and resuspended in DNAse-free water.

### HEKs cultivation and transfection

Human embryonic kidney (HEK) cell line 293 T (ATCC, CRL-3216) were cultured at 37°C (5% CO_2_) in HEK medium (DMEM high glucose + 0% Fetal Bovine Serum + 1% antibiotic–antimycotic). At 80% confluence, the colonies were detached using Trypsin-EDTA (Thermo Fisher Scientific, 0779413) and passaged in a 1:5 or 1:10 split ratio. To ensure cellular attachment, glass coverslips were coated with poly-ʟ-lysine before splitting. To transfect HEKs, the day after splitting, plasmidic DNA was mixed with Polyethyleneimine (PEI) Max (Kyfora Bio, 24885) in DMEM and incubated 15 min at room temperature. Cells were fixed or collected 24 h after transfection.

### Primary rat cortical neurons

Primary cultures of rat cortical neurons were prepared from rat embryos (Sprague–Dawley rats, Janvier Labs) at embryonic day 18 as previously described (Catanese et al., 2019). Cerebral cortices were manually dissected under stereomicroscopic guidance. After 10 min of incubation with 0.25% trypsin-EDTA, tissues were washed three times with DMEM F12 and resuspended in DMEM +++ (DMEM F12 containing 10% fetal bovine serum, 1% penicillin/streptomycin, and 1% GlutaMAX) and thus mechanically dissociated. Following a filtration through a 100 µm mesh filter, the dissociated cells were plated on poly-ʟ-lysine–coated (Sigma-Aldrich) glass coverslips or plastic dishes. Two hours after plating, culture medium was changed to Neurobasal +++ (Neurobasal medium Invitrogen containing 0.5% Pen/Strep, 1% GlutaMAX, and 2% B27).

To overexpress KIF5A constructs, primary neurons were washed three times with DMEM F12 and medium was changed to Neurobasal ++ (Neurobasal medium Invitrogen containing 1% GlutaMAX and 2% B27) to remove any trace of antibiotic in the culture. DNA was mixed with Optifect transfection reagent (Thermo Fisher Scientific, 12579017) in DMEM for 20 min at room temperature. Neurons were incubated with the solution for 5 h at 37°C. After incubation time, cells were washed three times with DMEM F12 and cultured in a solution of Neurobasal +++ and conditioned medium (1:1 ratio) previously collected. Neurons were fixed after 1 or 2 d of transfection.

### Transmission electron microscopy

To perform TEM analysis, samples were fixed by high-pressure freezing as previously described (Catanese et al., 2018; Walther et al., 2018). Cells were cultivated onto sapphire discs (Engineering Office M. Wohlwend), and at the indicated time point of cultivation, the sapphire discs were removed and dipped in 95% 1-hexadecene (Merck Millipore, 822064). Two sapphire discs oriented face to face and separated by a gold ring (Plano, G2620A; 3.05 mm diameter) were mounted into a holder (Engineering Office M. Wohlwend) and placed into a Wohlwend HPF Compact 01 high-pressure freezer (Engineering Office M. Wohlwend). The samples were frozen with liquid nitrogen at a pressure of 2,100 bar. After high-pressure freezing, the sapphire discs were separately incubated in 1.5 ml precooled (−87°C) sample tubes filled with 1 ml of freeze substitution solution (0.2% osmium tetroxide, 0.1% uranyl acetate, 5% dH_2_O in acetone). After the tube had been warmed up to 0°C, samples were washed three times with 1 ml of pure acetone and transferred into a clean tube containing 0.25 ml of pure epoxy resin (Honeywell Fluka, 45345-1L-F) and incubated for 24 h at 60°C to polymerize. The samples were stored at room temperature after polymerization.

For specimen preparation, 70–100-nm-thick sections were cut off from the epoxy resin block parallel to the plane of the sapphire disc with an Ultracut UCT ultramicrotome (Leica) equipped with a diamond knife (Science Services). After mounting the slice onto a 300-mesh copper grid (Plano, G2300C-M), sections were stained with 0.3% lead citrate for 1 min, washed with dH_2_O, and dried at RT. The samples were examined with a Jeol JEM 1400 (Jeol) transmission electron microscope at 100 kV. Images were randomly recorded with an exposure time of 14,000 ms and saved with a resolution of 4 megapixels (2.048 × 2.048). Images were then manually analyzed using ImageJ. Aggresomes were identified as large, nonperfectly round-shaped and membrane-bound structures enclosing in their lumen membrane and organelle residuals, as well as electron-dense material (Klionsky et al., 2016). We considered aggresomes only in those structures with a minimum size of 0.250 µm^2^. The size of every aggresome was then measured and plotted.

### Immunocytochemistry

Immunocytochemistry (ICC) was performed as previously described (Catanese et al., 2021). Following fixation with 4% paraformaldehyde containing 10% sucrose, cells were first incubated with blocking solution (PBS + 10% Donkey Serum + 0.2% Triton X-100) for 1 h at 4°C and subsequently with primary antibodies diluted in the same blocking solution overnight at 4°C. After three washes with PBS, cells were incubated for 2 h at room temperature with secondary antibodies or with phalloidin Alexa Fluor 568 and diluted in blocking solution 1:1,000 and 1:40, respectively. Lastly, cells were washed again three times and mounted with ProLong Gold Antifade Mountant (Invitrogen, P36934) or ProLong Gold Antifade Mountant with DAPI (Invitrogen, P36935) and Ibidi Mounting Medium (Ibidi, 5001).

### Microscopy and image analysis

Confocal microscopy was performed with a laser scanning microscope (Leica DMi8) equipped with an ACS APO 63× oil DIC immersion objective. Images were acquired using the LAS X software (Leica), with a resolution of 1,024 × 1,024 pixels and a variable number of *Z*-stacks (step size of 0.3 µm) encompassing the entire cell soma.

Images were analyzed with ImageJ Fiji (Schindelin et al., 2012) software. For each cell, regions of interest (ROIs) were drawn to delimit the cytosol and nucleus. Mean intensity of the signals was measured within the ROI. To assess KIF5A protein accumulation in hiPSC-derived motoneurons, cytosolic ROIs were analyzed with FindFoci plugin (Herbert et al., 2014). Cytoplasmic TDP-43 mislocalization was quantified by measuring total TDP-43 fluorescence intensity in the cells, subtracting the signal corresponding to nuclear TDP-43 and normalizing it over total TDP-43 signal (Casiraghi et al., 2025). In case of aggregation analysis, a defined intensity threshold was applied to the GFP channel of each image to filter out background signals. The area occupied by the resulting fluorescence was divided to the cytosolic area defined by phalloidin (for hEKs) or MAP2 (for neurons) staining. Area ratios were then normalized on the average ratio of WT transfected cells.

### Data and statistical analysis

Statistical analyses were performed with GraphPad Prism (version 9.4.1) software. Data are presented as fold change relative to the respective control/baseline (if not differently stated). Normal distributions were assessed by Shapiro–Wilks test. Statistical significance was set at *p* < 0.05.

For Western blot analysis, values were normalized against the loading control, β-actin. One-tailed, paired tests were used to compare two populations: Paired *t* test as parametric test and Wilcoxon matched pair test as nonparametric. To confront KIF5A^c2993-1^ II versus KIF5A^c2993-1^ I (Fig. 2*C*), one-tailed Mann–Whitney (unpaired test, nonparametric) was employed. For immunostaining analysis, intensity values were normalized on the average intensity of the KIF5A WT transfected cells and on the average of not transfected cells for endogenous KIF5A while each ALS cell line was compared with its isogenic control. Unpaired, two-tailed tests were used to compare two populations: *t* test as parametric test and Mann–Whitney as nonparametric. For three or more populations, ordinary one-way ANOVA, followed by Dunett's multiple-comparison test, was used and Kruskal–Wallis, followed by Dunn's multiple-comparison test, for non-normally distributed populations. Sizes of structures identified by FindFoci were plotted as cumulative distributions to better display larger protein accumulations in ALS cell lines. In the aggregation analysis, the normalized area ratios were plotted as cumulative distribution to highlight the aggregation behavior of KIF5A constructs. Two-tailed Kolmogorov–Smirnov was used to compare two cumulative distributions and Kruskal–Wallis, followed by Dunn's multiple-comparison test, for three or more. More information can be found in the figure legends.

### Ethics approval

All procedures with hiPSC material were in accordance with the ethical committee of the Ulm University (Nr.0148/2009 and 265/12) and in compliance with the guidelines of the Federal Government of Germany. All participants gave informed consent for the study. The use of human material was approved by the Declaration of Helsinki concerning Ethical Principles for Medical Research Involving Human Subjects.

The preparation of rat primary neurons was allowed by the Permit Nr. O.103 of Land Baden-Württemberg (Germany) and performed in respect of the guidelines for the welfare of experimental animals issued by the German Federal Government and the Max Planck Society, and the ARRIVE guidelines.

## Results

### Mutant KIF5A neurons accumulate the aberrant KIF5A protein and cytosolic TDP-43

We reprogrammed iPSCs from the keratinocytes of an ALS patient carrying the c2993-1 G > A variant (KIF5A^c2993-1 G>A^) within the *KIF5A* gene. These cells were compared with an isogenic control generated by correcting the pathogenic mutation with CRISPR-Cas9 (KIF5A^WT^ I). We also included in our study a second pair of isogenic hiPSC lines generated by inserting the c2993-1 G > A mutation (KIF5A^c2993-1^ II) in the *KIF5A* gene of the commercial KOLF2.1J cell line (henceforth KIF5A^WT^ II).

**Figure 1.**
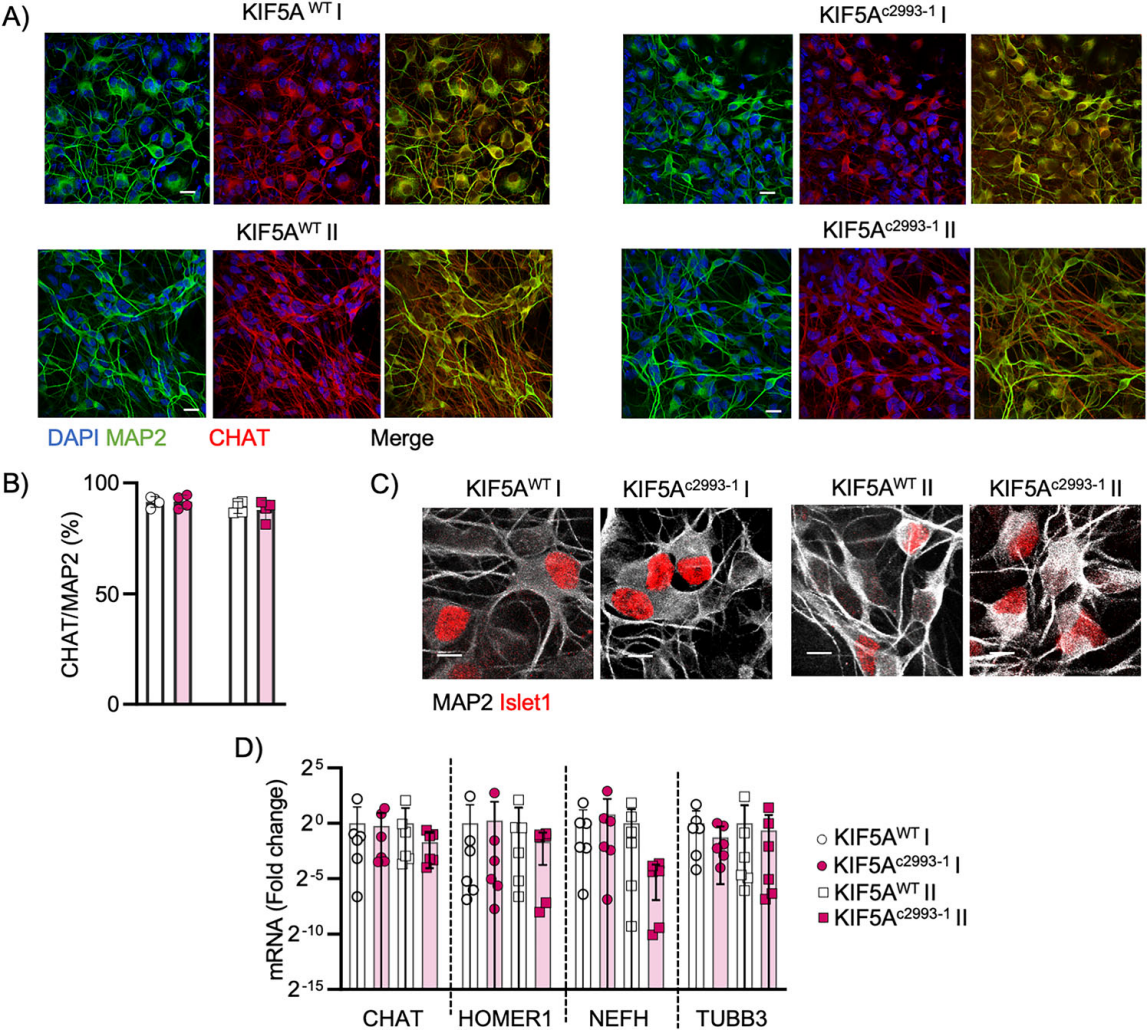
The KIF5A^c2993-1^ mutation does not affect the differentiation of hiPSC into MNs. ***A***, Representative images of hiPSC-derived neurons at DIV 21 (scale bar, 10 µm). In blue, DAPI; in green, MAP2; and in red, CHAT. ***B***, Most of motoneurons in the cultures were CHAT positive. *N* = 4 independent differentiations. Data plotted as mean ± SD, mean of KIF5A^WT^ I = 92%, KIF5A^c2993-1^ I = 91%, KIF5A^WT^ II = 89%, and KIF5A^c2993-1^ II = 88%. ***C***, Representative immunofluorescence images of hiPSC-derived motoneuron cultures at DIV 21 (scale bar, 10 µm) expressing Islet1 (in red). ***D***, All cell lines expressed cholinergic neuronal marker CHAT, synaptic marker Homer1, and neuronal markers Neurofilament heavy (NEFH) and Tubulin β-3 (TUBB3). *N* = 6 independent differentiation. Data plotted as mean ± SD and analyzed with two-way ANOVA.

Both isogenic pairs were efficiently differentiated into neurons following a previously described protocol (Catanese et al., 2021). Following this methodology, we obtained cultures that, at day in vitro (DIV) 21, were homogeneously characterized across genotypes by 60% of neurons, with 90% of them being CHAT-positive motoneurons (MNs; Fig. 1*A*,*B*), as also confirmed by the expression of the nuclear marker Islet1 (Fig. 1*C*). Notably, the presence of the pathogenic *KIF5A* mutation did not affect the differentiation and the expression of the neuronal markers CHAT, Homer1, Neurofilament heavy chain (NEFH), and Tubulin β-3 (TUBB3; Fig. 1*D*). Accordingly, the mutant lines did not show any significant increase in the ratio between the protein levels of the apoptotic marker cleaved caspase 3 and its full length in comparison with their corresponding isogenic control (Fig. S1). This indicates that KIF5A mutations do not impact neuronal differentiation and survival in DIV21 cultures.

Since ALS is a late-onset disease, we cultured KIF5A^c2993-1 G>A^ and KIF5A^iso^ neurons for nine weeks and focused on the appearance of pathology hallmarks. First, we investigated the expression of the wild-type (WT) and mutant forms of the KIF5A protein in both genotypes. To do this, we generated a custom-made antibody selectively raised against the aberrant C terminal of the protein linked to ALS. The specificity of this antibody was confirmed by the overexpression of full-length WT and mutant KIF5A (KIF5A-ALS henceforth) constructs in HEK cells, which do not endogenously express this neuron-specific protein. The mutant form of KIF5A was selectively detected by the custom-made antibody upon overexpression of the related construct, while the WT was recognized by a commercial antibody raised against the native C terminal. According to the mutation modifying only this terminal portion of KIF5A, a second commercial antibody raised against its Stalk domain could detect both forms of the protein (Fig. S2).

**Figure 2.**
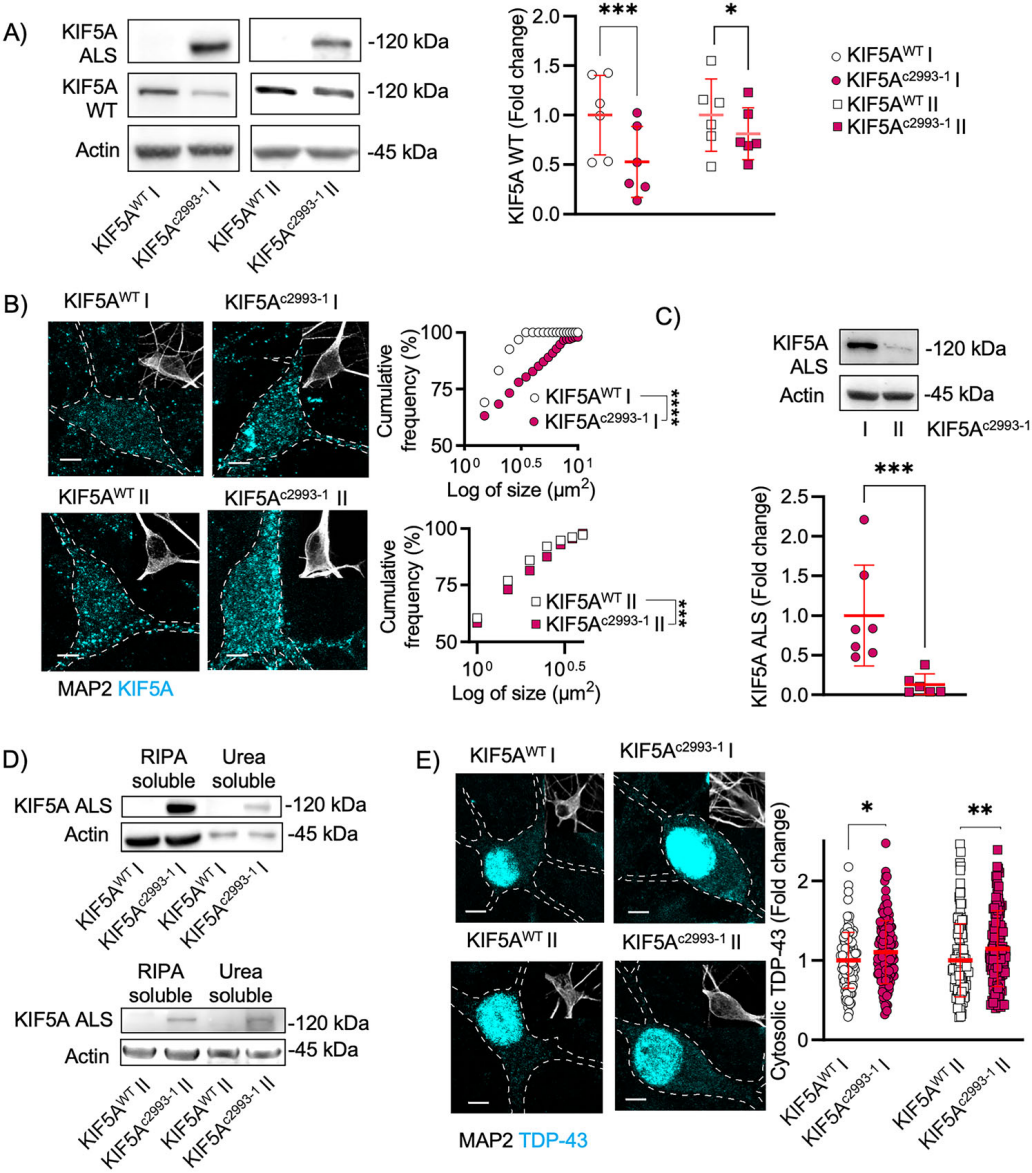
TDP-43 is mislocalized in hiPSC-derived motoneurons from ALS cell lines. ***A***, Representative immunoblots of hiPSC-derived neurons at DIV 63 showed a significant reduction of the WT isoform of KIF5A in the ALS cell lines. *N* = 6 independent differentiations. Data plotted as mean ± SD and compared by paired *t* test one-tailed. For KIF5A^c2993-1^ I versus KIF5A^WT^ I: ***p = 0.0007, mean of differences = −0.4732; for KIF5A^c2993-1^ II versus KIF5A^WT^ II: *p = 0.0352, mean of differences = −0.1885. ***B***, Representative immunofluorescence images of hiPSC-derived neurons (DIV 63; scale bar, 5 µm). ALS motoneurons displayed significantly larger KIF5A structures compared with their isogenic controls. Data from two independent differentiation plotted as cumulative frequency distributions (number of bins = 27; median of KIF5A^WT^ I = 1.285, mean of KIF5A^c2993-1^ I = 1.285, median of KIF5A^WT^ II = 1.028 and median of KIF5A^c2993-1^ II = 1.208) and analyzed with Kolmogorov–Smirnov, two-tailed. For KIF5A^c2993-1^ I versus KIF5A^WT^ I: ****p < 0.0001; for KIF5A^c2993-1^ II versus KIF5A^WT^ II: ***p = 0.0006. ***C***, Representative immunoblot of the pathological KIF5A isoform in ALS motoneurons (DIV 63). KIF5A^c2993-1^ II showed an 87% reduction of KIF5A-ALS expression compared with KIF5A^c2993-1^ I. *N* = 7 (KIF5A^c2993-1^ I) and 6 (KIF5A^c2993-1^ II) independent differentiations. Data plotted as mean ± SD; and compared with Mann–Whitney test, one-tailed. KIF5A^c2993-1^ II versus KIF5A^c2993-1^ I: ***p = 0.00006; differences between medians = −0.7535. ***D***, RIPA-insoluble fractions of cell lysates from mutant cultures contain the pathological isoform of KIF5A. ***E***, Representative immunofluorescence images of hiPSC-derived neurons (DIV 63; scale bar, 5 µm). Immunofluorescence analysis revealed TDP-43 (in cyan) cytosolic enrichment in ALS cell lines. *N* = 110 (KIF5A^WT^ I), 129 (KIF5A^c2993-1^ I), 125 (KIF5A^WT^ II), and 134 (KIF5A^c2993-1^ II) neurons from six independent differentiations. Data plotted as mean ± SD and compared with Mann–Whitney test, two-tailed. For KIF5A^c2993-1^ I versus KIF5A^WT^ I: *p = 0.0461; difference between medians = 0.0521; for KIF5A^c2993-1^ II versus KIF5A^WT^ II: ***p = 0.0056; difference between medians = 0.2160.

Using the custom-made antibody, we could detect the presence of mutant KIF5A in neurons differentiated from KIF5A^c2993-1^ iPSCs but not in the isogenic controls. Moreover, the levels of the endogenous protein with the physiological C terminal were significantly reduced in the mutant cells when compared with KIF5A^WT^ ones (Fig. 2*A*).

We then investigated whether mutant MNs might display an aberrant accumulation of the mutant KIF5A protein, as suggested by previous reports (Baron et al., 2022; Pant et al., 2022). By comparing the mutant lines to their corresponding isogenic controls, we found that the size of KIF5A-positive structures was significantly increased in the presence of the pathogenic mutation. Notably, while the neurons obtained from the patient hiPSCs displayed aberrant cytosolic KIF5A aggregates, the cells generated by inserting the c2993-1 variant with CRISPR-Cas9 were characterized by only a minor enlargement of KIF5A structures in comparison with the parental line (Fig. 2*B*). We speculated that this difference might be linked to the levels of the mutant protein itself and, indeed, we found that the KIF5A^c2993-1^ I MNs (obtained from the ALS patient) had significantly higher load of the mutant protein than the KIF5A^c2993-1^ II ones (generated using CRISPR-Cas9; Fig. 2*C*). Despite this incongruency, we found a portion of the mutant KIF5A protein within the urea-soluble fraction of the cultures from both lines, supporting the theory of aggregating KIF5A as part of the pathological features characterizing these ALS cases (Fig. 2*D*). In addition, we observed that the mutant MNs displayed a significant accumulation of cytosolic TDP-43 in comparison with the isogenic cells (Fig. 2*E*), suggesting a possible pathological synergism between the accumulation mutant KIF5A and TDP-43 pathology.

### Restoring the physiological isoelectric point of the C terminal prevents KIF5A aggregation

**Figure 3.**
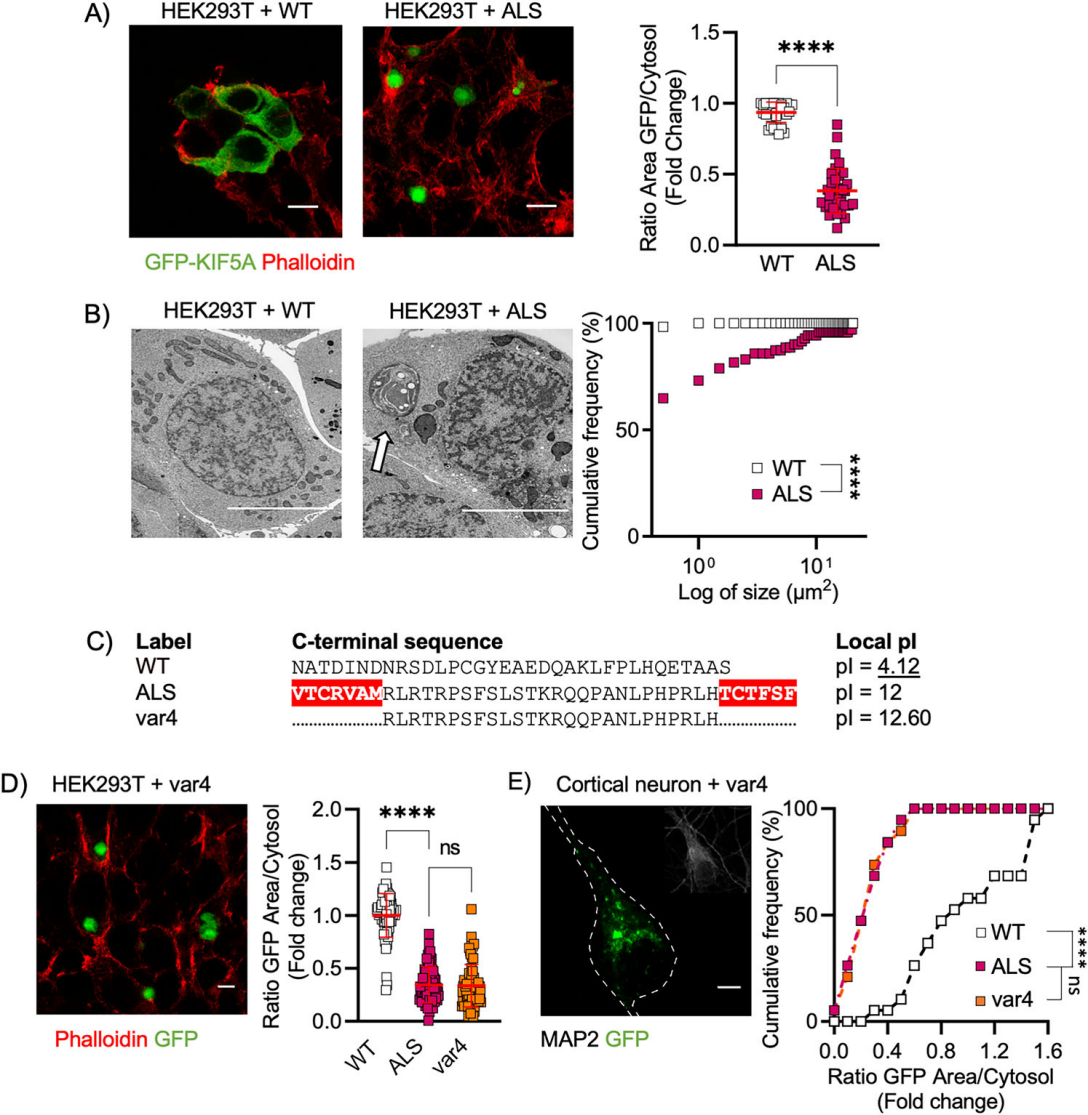
KIF5A-ALS aggregation in HEK depends on aberrant Cterm sequence's pI. ***A***, Representative immunofluorescence images of transfected HEKs (scale bar, 10 µm). The overexpression of the KIF5A-ALS construct led to protein aggregation. *N* = 32 (WT) and 36 (ALS) cells from three independent cellular preparations. Data plotted as mean ± SD and compared with Mann–Whitney test, two-tailed. ****p < 0.0001; difference between medians = 0.62. ***B***, Representative TEM images showed larger aggresomes in HEKs transfected with KIF5A-ALS construct (scale bar, 5 µm). Data from three independent cellular preparations plotted as cumulative frequency distributions (number of bins = 42, median of WT = 0.113, and median of ALS = 0.423) and analyzed with Kolmogorov–Smirnov, two-tailed, ****p < 0.0001. ***C***, Terminal aminoacidic sequences of the KIF5A WT, ALS and var4 constructs. The two aggregation-prone sequences identified by Aggrescan in the ALS sequence are highlighted in red. ***D***, Representative images of HEK (scale bar, 5 µm) transfected with KIF5A var4. Eliminating the two aggregation-prone sequences did not ameliorate protein aggregation. *N* = 66 (WT), 69 (ALS) and 68 (var4) cells from three independent cellular preparations. Data plotted as mean ± SD and analyzed with Kruskal–Wallis test followed by Dunn's multiple-comparison test to confront each condition to var4. For var4 versus WT: ****p < 0.0001, mean rank difference = 98.89; for var4 versus ALS: *p* > 0.999, mean rank difference = 4.488. ***E***, Representative image of primary cortical neurons (DIV 8; scale bar, 5 µm) transfected with KIF5A var4. As shown in HEKs, KIF5A var4 led to protein aggregation. Data from three independent cell preparation plotted as cumulative distributions (number of bins = 17, median of WT = 0.875, mean of ALS = 0.272, mean of var4 = 0.255) and analyzed with Kruskal–Wallis test, two-tailed, followed by Dunn's multiple-comparison test to confront each condition to var4. For var4 versus WT: ****p < 0.0001, mean rank difference = −27.21; for var4 versus ALS: *p* > 0.999, mean rank difference = −0.105.

We then aimed at investigating the biochemical features contributing to the aggregation of the KIF5A-ALS protein. To this end, we first overexpressed mutant KIF5A-ALS in HEK cells and observed the accumulation of aberrant aggregates in immunofluorescence (Fig. 3*A*) and high-pressure freezing electron microscopy (Fig. 3*B*). In order to clarify the aggregation behavior of mutant KIF5A, we used the Aggrescan tool (Conchillo-Solé et al., 2007) and identified two hotspots within the aberrant C terminal that were predicted to significantly contribute to KIF5A aggregation (Fig. 3*C*). Interestingly, deleting both sequences from the mutant C terminal was not sufficient to revert the distribution of KIF5A-ALS, which kept on aggregating even in this modified form when overexpressed in HEK cells (Fig. 3*D*) and in primary cortical neurons (Fig. 3*E*).

**Figure 4.**
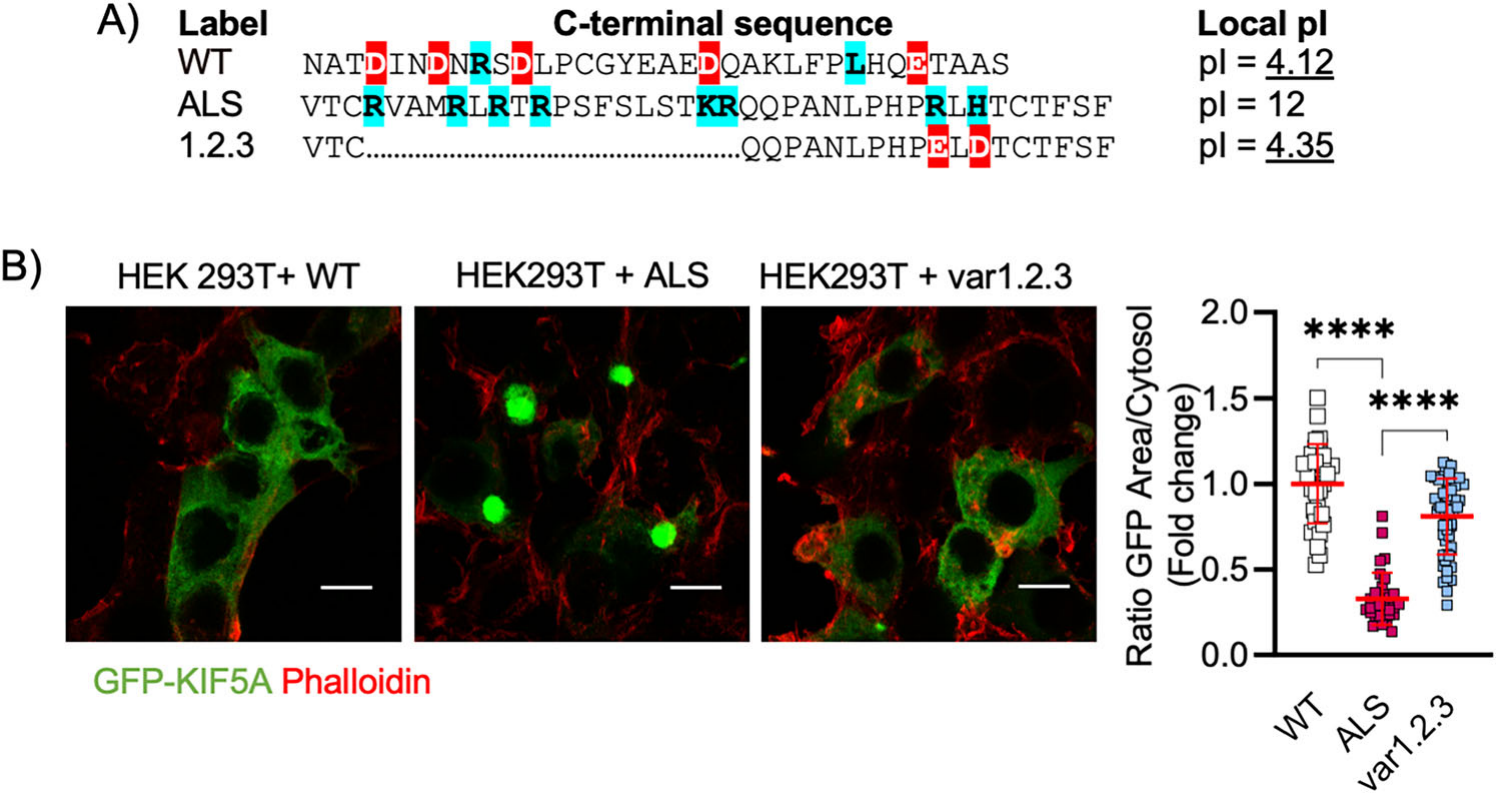
The pI of its C terminal contributes to KIF5A aggregation. ***A***, Aminoacidic sequences of KIF5A isoforms and their respective pI. Basic amino acids are labeled in blue while the acid ones in red. ***B***, Representative images of transfected HEKs. The variant 1.2.3 showed to have a homogenous cytosolic distribution similar to WT. *N* = 32 (WT), 32 (ALS), and 51 (var1.2.3) cells from three independent cellular preparations. Data plotted as mean ± SD and analyzed with Kruskal–Wallis test, two-tailed, followed by Dunn's multiple-comparison test to confront each condition with ALS. For ALS versus WT: ****p < 0.0001, mean rank difference = −168.2; for ALS versus var1.2.3: ****p < 0.0001, mean rank difference = −136.

Since folding and aggregation are influenced by the protein isoelectric point (pI), which is largely determined by the aminoacidic charge, we focused on this biochemical aspect. As previously observed by Baron and colleagues, the pI of the KIF5A C terminal undergoes a switch from 4.12 (WT sequence) to 12 in the ALS sequence, which appears to be determined by the presence of eight basic amino acids in the mutant peptide. Thus, we reasoned that re-establishment of a pI close to the one of the WT C terminal might prevent KIF5A from aggregating. We used site-directed mutagenesis to introduce several modifications within the C terminal of KIFA-ALS by either exchanging single amino acids or deleting portions of the aberrant sequence to reduce its pI. The substitution of single basic amino acid with acid ones had only a marginal effect on the ALS C-terminal pI (variants 2, 3, and 2.3; Fig. S3*A*), and even if the deletion of a 18 aa sequence from the mutant C terminal was more efficient in bringing the pI to lower values (variants 1, 1.2, and 1.3; Fig. S3*A*), these were still higher than 5 and all the KIF5A variants formed aberrant aggregates in HEK cells (Fig. S3*B*). Notably, when we replaced the two basic amino acids (arginine and histidine) still present in the ALS C terminal of the variant 1 (where the 18 aa sequence enriched in basic amino acids was deleted) with a glutamic and aspartic acid (variant 1.2.3), respectively, the pI of the aberrant peptide reached a value of 4.35 close to the one of the WT protein (Fig. 4*A*, Fig. S3*B*) and, according to our theory, this variant did not display any aggregation behavior (Fig. 4*B*).

### Mutant C terminal with an isoelectric point similar to the WT prevents KIF5A from aggregating and does not trigger degenerative downstream phenotypes in neurons

**Figure 5.**
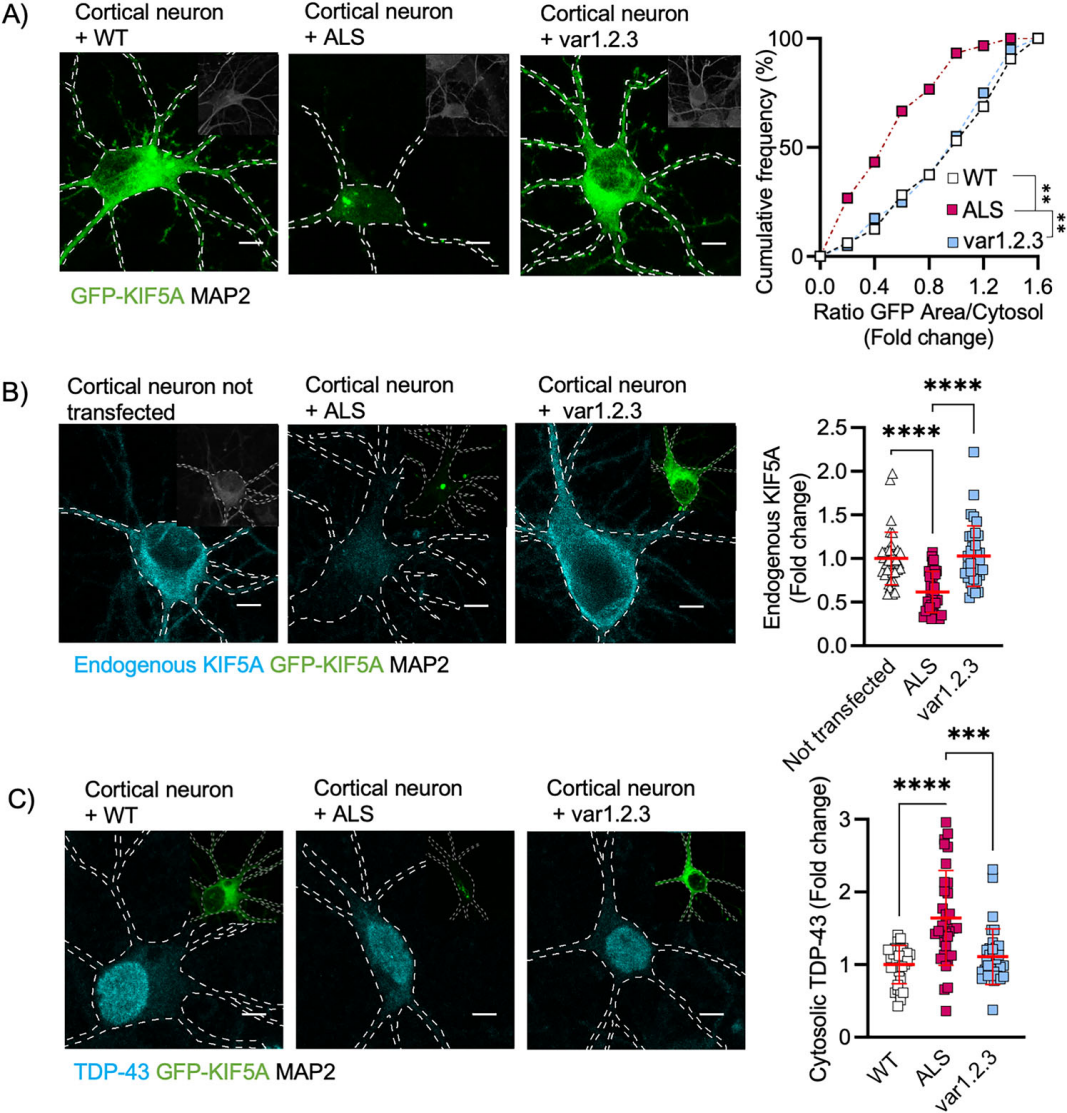
Restoring the original pI ameliorates TDP-43 mislocalization and endogenous KIF5A in primary neurons. ***A–C***, Representative images of transfected primary cortical neurons (DIV 8; scale bar, 5 µm). ***A***, KIF5A variant 1.2.3 showed lower aggregation behavior than ALS. Data from three independent cellular preparations plotted as cumulative frequency distributions (number of bins = 9, median of WT = 1.067, median of ALS = 0.55, and median of var1.2.3 = 1.048) and analyzed by Kruskal–Wallis test, two-tailed, followed by Dunn's multiple-comparison test to confront each condition to ALS. For ALS versus WT: **p = 0.001, mean rank difference = −41.74; for ALS versus var1.2.3: **p = 0.001, mean rank difference = −39.09. ***B***, In cyan, staining for endogenous KIF5A. Overexpression of KIF5A-ALS led to lower levels of endogenous KIF5A compared with untransfected neurons and neurons overexpressing the variant 1.2.3. *N* = 36 (not transfected), 34 (ALS), and 35 (var1.2.3) primary cortical neurons from three independent cell preparations. Data plotted as mean ± SD and analyzed with Kruskal–Wallis test, two-tailed, followed by Dunn's multiple-comparison test to confront each condition to ALS. For ALS versus not transfected: ****p < 0.001, mean rank difference = −36.75; for ALS versus var1.2.3: ****p < 0.0001, mean rank difference = −37.20. ***C***, In cyan, staining for TDP-43. KIF5A-ALS overexpression led to higher cytosolic TDP-43 compared with WT and var1.2.3 overexpression. *N* = 31 (WT), 32 (ALS), and 33 (var1.2.3), 29 (var1), and 24 (var1.2) primary cortical neurons from three independent cellular preparations. Data plotted as mean ± SD and analyzed with Kruskal–Wallis test, two-tailed, followed by Dunn's multiple-comparison test to confront each condition to ALS. For ALS versus WT: ****p < 0.0001, mean rank difference = 49.67; for ALS versus var1.2.3: ***p = 0.0003, mean rank difference = 42.58; for ALS versus var1: *p* > 0.999, mean rank difference = 19.70 and for ALS versus var1.2: *p* > 0.999, mean rank difference = 28.88.

**Figure 6.**
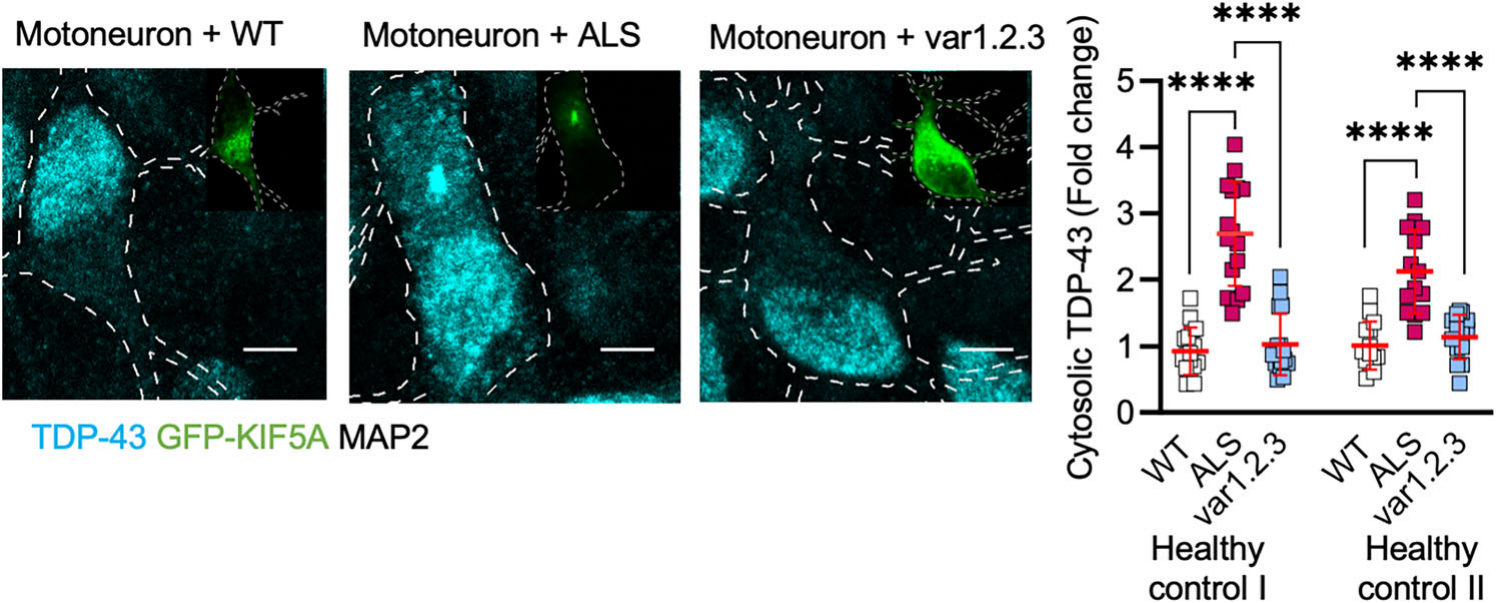
KIF5A-ALS overexpression in control motoneurons resulted in TDP-43 mislocalization and sequestration in KIF5A aggregates. In cyan, TDP-43 in transfected healthy control motoneurons (DIV 21; scale bar, 5 µm). KIF5A-ALS overexpression led to cytosolic mislocalization and aggregation of TDP-43. For Healthy control I: *N* = 16 (WT), 17 (ALS), and 16 (var1.2.3) neurons from three independent differentiations. Data plotted as mean ± SD and analyzed with Kruskal–Wallis test, two-tailed, followed by Dunn's multiple-comparison test to confront each condition to ALS. For ALS versus WT: ****p < 0.0001, mean rank difference = 36.43; For ALS versus var1.2.3: ****p < 0.0001, mean rank difference = 34.06. For Healthy control II: *N* = 14 (WT), 14 (ALS), and 14 (var1.2.3) neurons from three independent differentiations. Data plotted as mean ± SD and analyzed with one-way ANOVA, two-tailed, followed by Dunnett's multiple-comparison test to confront each condition to ALS. For ALS versus WT: ****p < 0.0001, mean difference = 1.107; for ALS versus var1.2.3: ****p < 0.0001, mean difference = 0.98.

We then tested whether the overexpression of the KIF5A-ALS variant 1.2.3 might be less effective in inducing pathological phenotypes in neuronal cells. As observed in HEKs, the KIF5A-ALS protein, as well as other variants with higher pI, formed toxic aggregates in primary cortical neurons (Fig. S4), but these aberrant structures were not detectable in the case of the modified variant 1.2.3 with pI of 4.35 (Fig. 5*A*). Notably, the presence of KIF5A-ALS aggregates also significantly reduced the levels of endogenous KIF5A, when compared with untransfected neurons (Fig. 5*B*), resembling what was observed in patients’ neurons as consequence of the genetic mutation, and the endogenous levels of KIF5A in neurons expressing the variant 1.2.3 were comparable with those of nontransfected cells (Fig. 5*B*). Since the modified variant of KIF5A-ALS was not recognized by the antibody binding to the native C terminal (Fig. S5), this strengthened the idea that toxic aggregates might be required for a pathological sequestration of the WT KIF5A, which occurs through the interaction between the two forms of the protein and contributes to neuronal degeneration (Hares et al., 2021). In fact, in contrast to the KIF5A-ALS full length, overexpression of the KIF5A variant 1.2.3 did not induce any cytosolic accumulation of TDP-43 in primary cortical neurons (Fig. 5*C*), as well as in human MNs differentiated from control hiPSCs, which even displayed TDP-43 aggregation and sequestration upon KIF5A-ALS overexpression (Fig. 6).

**Figure 7.**
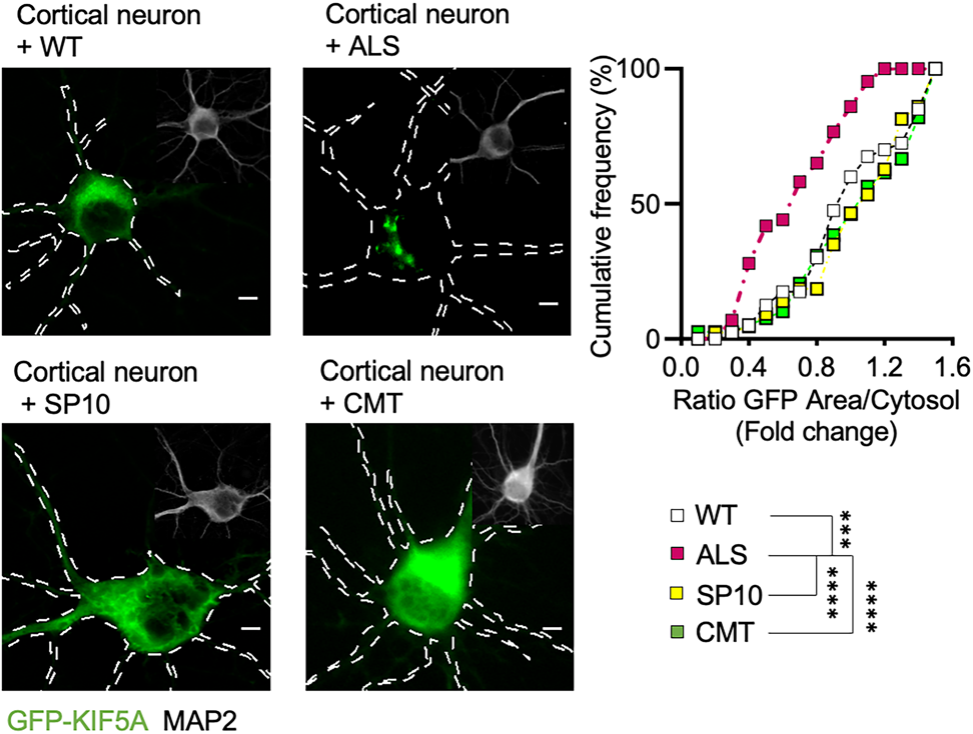
Mutations linked to SP10 and CMT do not induce KIF5A aggregation. Representative images of primary cortical neurons transfected with KIF5A isoforms (DIV 8; scale bar, 5 µm). Overexpression of CMT and SP10 constructs did not lead to protein aggregation. Data from three independent cell preparation plotted as cumulative distributions (number of bins = 16, median of WT = 0.96, median of ALS = 0.675, median of SP10 = 1.156, and mean of CMT = 1.082) and analyzed with Kruskal–Wallis test, two-tailed, followed by Dunn's multiple-comparison test to confront each condition to ALS. For ALS versus WT: ***p = 0.0004, mean rank difference = 40.22; for ALS versus SP10: ****p < 0.0001, mean rank difference = −49.67; for ALS versus CMT: ****p < 0.0001, mean rank difference = 49.80.

Thus, mutations in KIF5A leading to the generation of an aberrant C terminal with a pI higher than the WT are sufficient to cause KIF5A aggregation and TDP-43 cytosolic mislocalization, as typically observed in the ALS cases. In fact, *KIF5A* mutations linked to SP10 or CMT diseases do not alter its pI and did not lead to KIF5A aggregation in cortical neurons (Fig. 7).

## Discussion

Although the pathological mechanisms contributing to neuronal degeneration are numerous and heterogeneous, the accumulation of cytotoxic protein aggregates is one of the major hallmarks characterizing vulnerable cells. In ALS, the cytoplasmic mislocalization and aggregation of TDP-43 represents a common phenotype shared by the majority of the cases across the disease spectrum, with exception of SOD1 and FUS patients (Neumann et al., 2006). The aggregation of TDP-43 has been largely investigated since it mostly occurs in absence of mutations within the associated *TARDBP* gene (which count for <10% of the familial ALS cases; Kabashi et al., 2008; Sreedharan et al., 2008) and has been also observed in different neurological disorders such as frontotemporal dementia and Alzheimer's disease (Arai et al., 2006; Amador-Ortiz et al., 2007). From the structural point of view, TDP-43 contains a β-sheet-rich helical amyloid core which, upon protein cleavage, contributes to the formation of toxic aggregates (Kumar et al., 2023). These structures contain indeed the full-length protein as well as abnormally cleaved fragments (Neumann et al., 2006) and form as a consequence of impaired multimerization (Oiwa et al., 2023) or in response to different stress stimuli (Lee et al., 2022). Since vulnerable neurons are characterized by impaired physiological properties, which arise from the altered function of disease-causative genes, the accumulation of TDP-43 toxic species appears to be a convergent pathological feature indicative of cellular sufferance. Interestingly, and in agreement with the heterogeneity of ALS genes, the cellular and biochemical alterations leading to TDP-43 aggregation, are numerous and diverse. For example, sequestration of TBK1 within C9orf72-related polyGA aggregates impairs autophagy and induces TDP-43 phenotype (Shao et al., 2022), while reduced levels of NEK1 are also linked to the cytosolic accumulation of the RNA-binding protein, most likely due to a lower kinase activity (Rifai et al., 2024).

In the case of KIF5A, the mutant C terminal resulting from ALS mutations has a reduced interaction with its motor domain, which results in increased motility through impaired autoinhibition (Baron et al., 2022; Pant et al., 2022). The reduced stability of the autoinhibited state(Carrington et al., 2024) seems to promote KIF5A aggregation in presence of the aberrant 39-aa-long C terminal, which spans over an intrinsically disordered domain already in its WT sequence (Seeger et al., 2012). Notably, the aberrant C terminal has been described to induce aggregation of the purified KIF5A protein in vitro, indicating that its altered motility might not be the only factor leading to the formation of toxic oligomers (Nakano et al., 2022). Here we show that the switch linked to changes in the electric charge of the C terminal, which has a higher pI in its ALS variant, plays a key role in KIF5A aggregation. In fact, removing and/or exchanging the basic amino acids from the ALS C terminal prevents the formation of toxic protein structures. While the presence of these aggregates has already been shown to be toxic in different ALS models (Baron et al., 2022; Pant et al., 2022; Soustelle et al., 2023), our results highlighted two important aspects of the pathobiochemistry triggered by the accumulation of KIF5A aggregates that were previously unappreciated. In fact, the cytoplasmatic accumulation of TDP-43 was not described in previous work with ALS-KIF5A models, likely because of the preferential appearance of this phenotype in cells of the central nervous system. In fact, Pant and colleagues did not observe TDP-43 pathology upon overexpression of the ALS-related KIF5A ΔExon27 construct in HEK cells. While this pathological manifestation might be somehow expected in ALS neurons, the causal relation with mutant KIF5A being sufficient to induce TDP-43 mislocalizazion as a consequence of altered electric charge of its C terminal represents an important pathological aspect linked to this ALS gene. The altered electric charge of the KIF5A C terminal triggers indeed the formation of cytotoxic protein structures, whose cellular load appears to play a crucial role in inducing TDP-43 pathology. Indeed, the acute overexpression of the mutant KIF5A in the most vulnerable cells in ALS (spinal MNs) induced the aggregation of the RNA-binding protein and its sequestration within KIF5A structures (Fig. 6). In a more general perspective, the cytosolic mislocalization and subsequent aggregation of TDP-43 might represent a progressive manifestation linked to the levels of cellular stress displayed by the individual neurons. Vulnerable MNs might be indeed characterized by a threshold of resilience allowing them to resist to the pathological alterations occurring at the early stages of the disease. In the specific case of KIF5A, the time-dependent accumulation of the toxic mutant protein might contribute to determine the timings of neuronal sufferance and degeneration, which include TDP-43 pathology as observed in the majority of ALS cases.

All in all, our work highlights novel, detrimental biochemical aspects triggered by the pI switch occurring at the C terminal in the presence of ALS mutations, which starts a sequence of pathological events downstream KIF5A aggregation.

## Data Availability

All data supporting the findings of this study are contained within the article.
